# Glutathione facilitates enterovirus assembly by binding at a druggable pocket

**DOI:** 10.1038/s42003-019-0722-x

**Published:** 2020-01-03

**Authors:** Helen M. E. Duyvesteyn, Jingshan Ren, Thomas S. Walter, Elizabeth E. Fry, David I. Stuart

**Affiliations:** 1 Division of Structural Biology, University of Oxford, The Henry Wellcome Building for Genomic Medicine, Headington, Oxford OX3 7BN UK; 2 Diamond Light Source, Harwell Science and Innovation Campus, Didcot, OX11 0DE UK

**Keywords:** X-ray crystallography, Molecular medicine, Virus structures, Antivirals

## Abstract

Enteroviruses cause a range of human and animal diseases, some life-threatening, but there remain no licenced anti-enterovirus drugs. However, a benzene-sulfonamide derivative and related compounds have been shown recently to block infection of a range of enteroviruses by binding the capsid at a positively-charged surface depression conserved across many enteroviruses. It has also been established that glutathione is essential for the assembly of many enteroviruses, interacting with the capsid proteins to facilitate the formation of the pentameric assembly intermediate, although the mechanism is unknown. Here we show, by high resolution structure analyses of enterovirus F3, that reduced glutathione binds to the same interprotomer pocket as the benzene-sulfonamide derivative. Bound glutathione makes strong interactions with adjacent protomers, thereby explaining the underlying biological role of this druggable binding pocket and delineating the pharmacophore for potential antivirals.

## Abstract (web summary)

Helen Duyvesteyn et al. present a high-resolution structural analysis of gluthathione binding to an enterovirus capsid. They show that glutathione binds to a surface cavity, promoting assembly of the viral capsid, and providing a potential therapeutic target.

## Introduction

The genus *Enterovirus* (EV) is the most populous genus of the *Picornaviridae* family of small, single-stranded RNA viruses. Enteroviruses are responsible for a range of animal and human diseases. These non-enveloped viruses are constructed of an icosahedral protein capsid composed of 60 copies each of viral proteins VP1–4. VP1–3 are similar to each other (comprising a β-barrel with extended surface loops and termini) and form the capsid surface. Whilst a handful of enterovirus vaccines are available, including for poliovirus (PV) and for EV-A71 (frequently responsible for serious outcomes of hand, foot and mouth disease)^1–4^, there are as yet no licenced anti-enterovirus drugs, despite evidence for efficacy in clinical trials and reports of extremely high potency for compounds targeting a pocket internal to capsid protein VP1 (termed here the *P* site)^5,6^. However, a cavity on the capsid surface at an inter-protomer interface, where we previously reported extra electron density^7^, has recently been identified as potentially druggable, with micro-molar binding for a benzene-sulfonamide derivative^8^. This compound, and a number of related compounds, are inhibitors of Coxsackie B viruses and a range of other enteroviruses including rhinoviruses, and have been shown to act synergistically with *P* site binders^8^.

Cells contain glutathione in both reduced (GSH) and oxidised (GSSG) forms, whose balance is crucial for maintaining cellular redox potential^9^. Disruption of the GSH/GSSG balance is associated with several enterovirus infections, including EV-A71^10^, CV-A16 and CV-B3^11–13^. Furthermore, depletion of GSH, using l-butathione sulfoximate (BSO)^14,15^ and TP219^16^, blocks the assembly of protomeric units into pentamers^14,16,17^. GSH-independence can be conferred by mutations at the protomer interface, consistent with GSH binding to protomers driving the formation of pentameric units critical to assembly^16,17^. However, there are no published structural data for GSH binding to an enterovirus capsid.

Here we study EV-F3, a relatively benign bovine enterovirus which is our model system of choice^7,18–22^. We firstly show that EV-F3 is dependent on GSH and that GSH stabilises the capsid. We then determine high-resolution structures of complexes of GSH and its initial breakdown product Cys-Gly (CG) bound to EV-F3 and by a competition binding study demonstrate that GSH binds more strongly. We find that the binding site of both compounds is identical to that for a similar molecule that remains naturally attached to the virus^7^, and to the binding site observed for the benzene-sulfonamide derivative in complex with Coxsackievirus B3^8^, identifying the biological role for this binding site.

## Results

### EV-F3 is dependent on and stabilised by GSH

Infection of cells both with and without BSO treatment shows that inhibition of glutathione synthesis reduces EV-F3 growth by 3.5 log, demonstrating glutathione dependency (Supplementary Table 1). Since GSH binding has been shown to stabilise PV^17,23^, we next used the PaSTRy assay^24^ to demonstrate that GSH also shows a modest dose-dependent stabilisation of EV-F3 (Methods, Supplementary Fig. 1).

### GSH and CG bind at an inter-protomer surface pocket on EV-F3

**Fig. 1:**
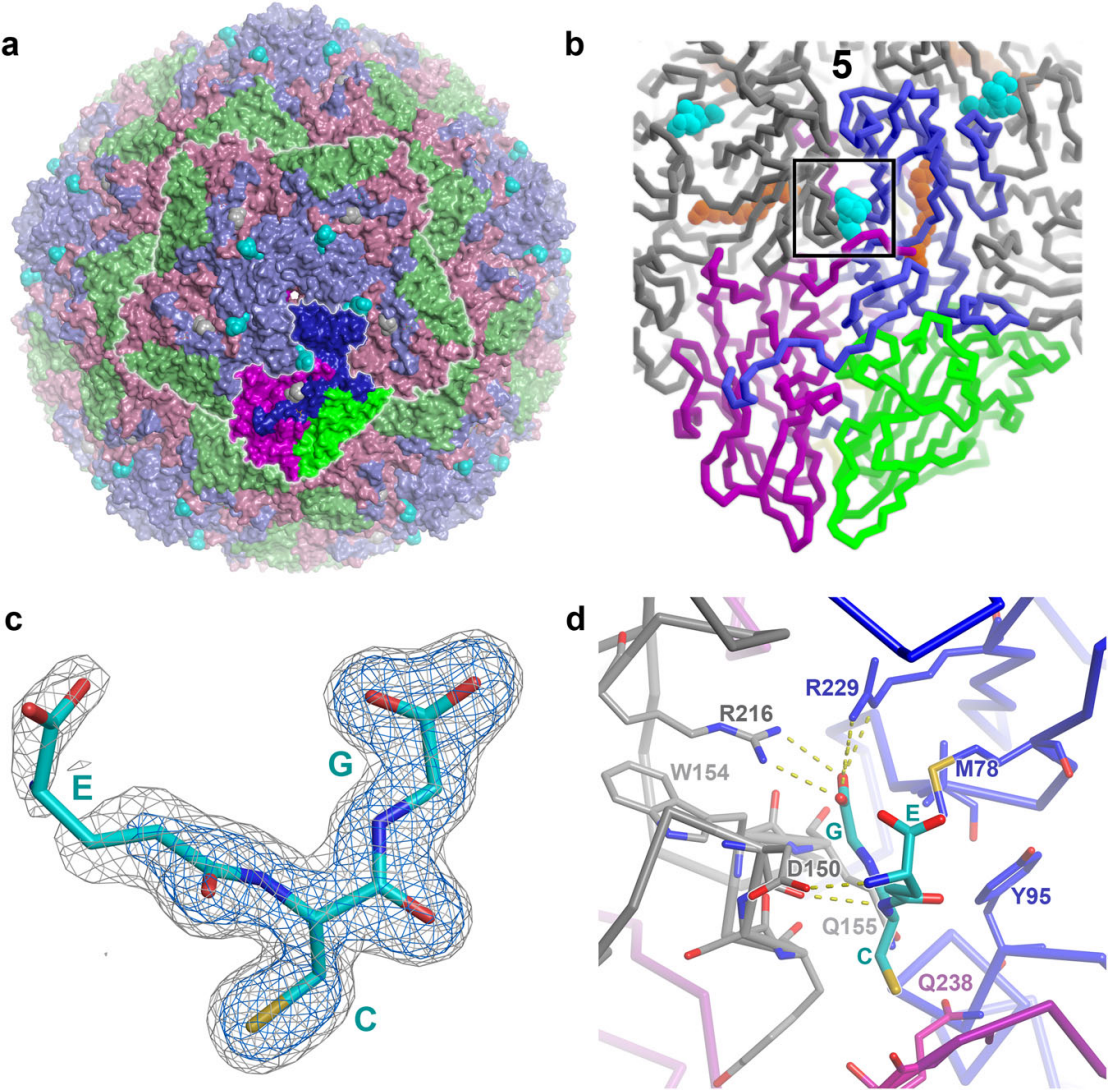
Virus−glutathione interactions. **a** EV-F3 capsid surface coloured by protein: VP1 blue, VP2 green, VP3 magenta. GSH at Site *I* is in cyan, and a weaker binding site is shown in white. A pentameric unit and a protomeric unit are both outlined in white and the protomer is coloured bold. **b** Cartoon of protomeric unit (with adjacent protomers shown in grey) with druggable sites: *P* orange, *I* cyan. Protein chains in protomeric unit A coloured as in (**a**), with VP4 in yellow (barely visible on the inside of the capsid). The position of the fivefold axis is marked “5”. **c** 15-fold NCS-averaged electron density for GSH, contoured at two levels, the blue level being 2.5 times higher. **d** GSH at site *I*. Protomer A is coloured as in (**b**), protomer B is in grey. The region shown in panel (**d**) is boxed on panel (**b**).

To determine if GSH binds to the enterovirus capsid, data to 1.8 Å resolution were collected from crystals of EV-F3 soaked with GSH by automated X-ray cryo-crystallography (Methods and Table 1), yielding high-quality electron density and a reliable model for surface-bound GSH (Fig. 1, Supplementary Fig. 2 and Table 1). GSH binds in a cavity between protomers within the pentameric building block (Fig. 1a–d), consistent with GSH’s role in protomer assembly^14,16^. We term this the interface (*I*) site. There is also an additional weak binding site nestled between the VP1 and VP3 C-termini at a non-conserved site, but this is probably not biologically significant (Fig. 1a).

**Table 1:** Data collection and refinement statistics.

	Experiment 1: EV-F3 GSH	Experiment 2: EV-F3 CG	Experiment 3: EV-F3 GSH/CG
Data collection			
Number of crystals	2	1	2
Space group	I222	I222	I222
Cell dimensions			
* a*, *b*, *c* (Å)	342.8, 348.2, 351.4	342.7, 348.3, 351.6	344.0, 349.4, 352.7
*α*, *β*, *γ* (°)	90, 90, 90	90, 90, 90	90, 90, 90
Resolution (Å)	20.0–1.80 (1.83–1.80)	20.0–1.67 (1.70–1.67)	110.0–2.17 (2.20–2.17)
* R*_merge_	0.394 (—)	0.216 (—)	0.374 (0.798)
* R*_pim_	0.111 (—)	0.089 (—)	0.187 (0.787)
* I*/σ*I*	6.2 (0.5)	7.2 (0.7)	5.7(3.6)
Completeness (%)	99.9 (99.9)	99.7 (95.4)	82.6 (76.9)
Redundancy	13.4 (7.8)	6.9 (5.2)	3.7(1.4)
Refinement			
Resolution (Å)	20.0–1.80	20.0–1.67	20.0–2.17
No. reflections	179,939/95,446	2,243,186/117,927	862,708/45,615
* R*_work_/*R*_free_	0.205, 0.208	0.190/0.191	0.200/0.203
No. atoms			
Protein	6291	6291	6288
Ligand/ion	86	79	94
Water	717	742	612
* B*-factors			
Protein	22.2	22.4	18.5
Ligand/ion	44.4	37.6	50.9
Water	31.1	34.9	33.8
R.m.s. deviations			
Bond lengths (Å)	0.005	0.005	0.005
Bond angles (°)	1.4	1.4	1.4

**Fig. 2:**
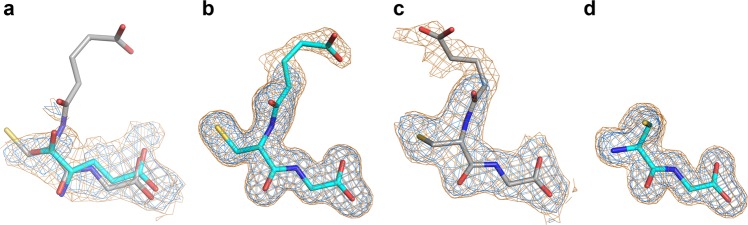
Electron density for GSH environments at the *I* site. **a** XFEL EV-F3 (PDB ID, 5OSN). **b** GSH soak (experiment 1). **c** competition soak (experiment 3). **d** CG soak (experiment 2). All maps are NCS-averaged and the view is the same for all panels. Two contour levels are shown, with blue 2.5 times higher than orange.

In un-soaked EV-F3 virions, the *I* site is partially occupied by a ligand similar to GSH (Fig. 2a) and to clarify the nature of the binding we performed two further structure determinations, firstly with CG (the GSH breakdown product) and secondly a competition soak with equal concentrations of both GSH and CG (Methods and Fig. 2). In summary, these experiments demonstrated that (i) GSH can replace the natural bound factor, (ii) that Cys-Gly (CG), the initial breakdown product of GSH^9^, also binds at the *I* site and (iii) that GSH binds more strongly than CG (Fig. 2, Methods and Table 1).

**Fig. 3:**
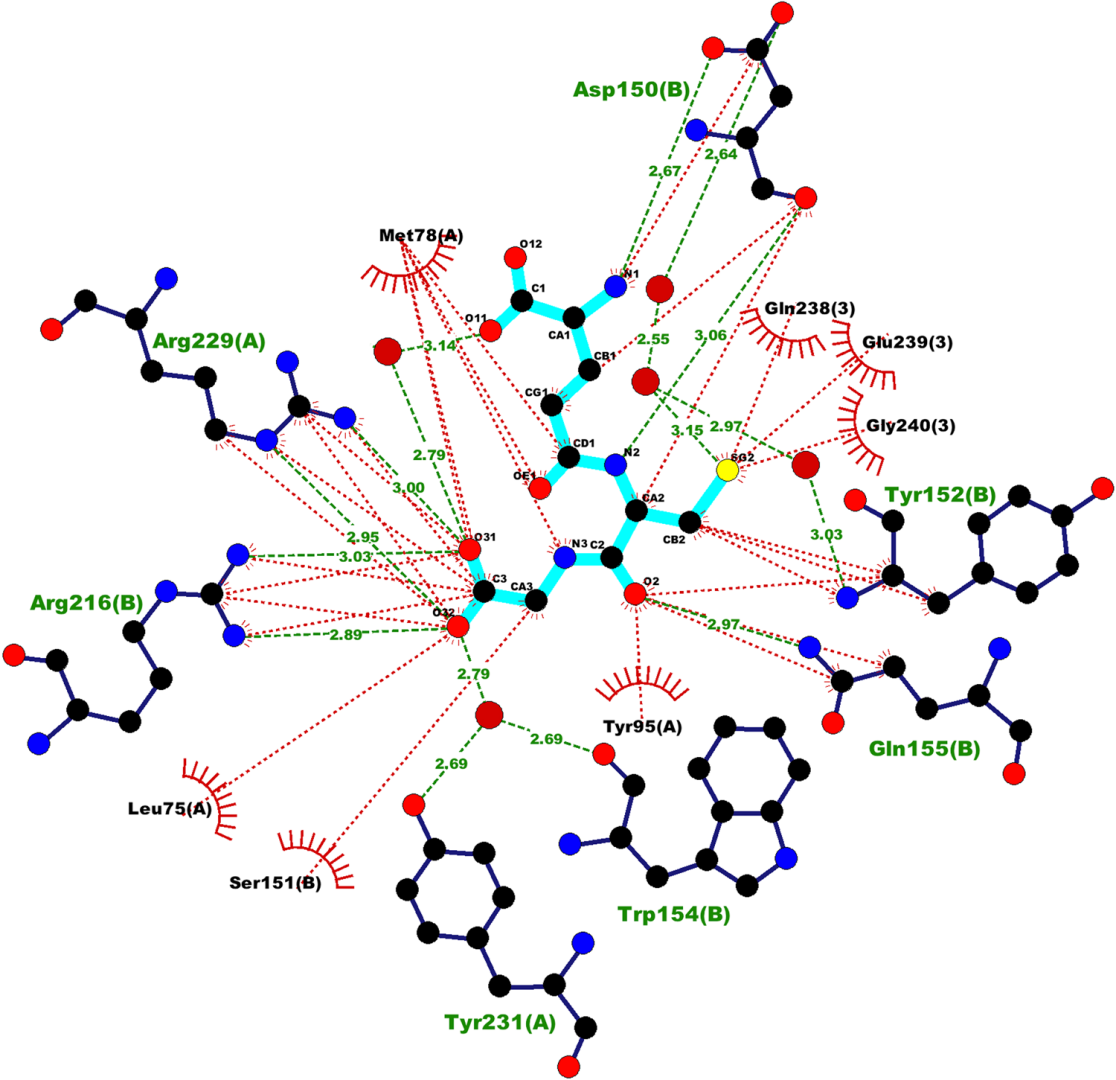
Details of interactions of GSH at the *I* site. GSH is shown in cyan. Red arcs denote hydrophobic interactions. The residue numbers for the interacting protein residues are shown with the chain label in parentheses (**A** is VP1 of protomer A, **B** is VP1 of protomer B and **3** is VP3 of protomer A). Drawn with Ligplot+^41^.

**Fig. 4:**
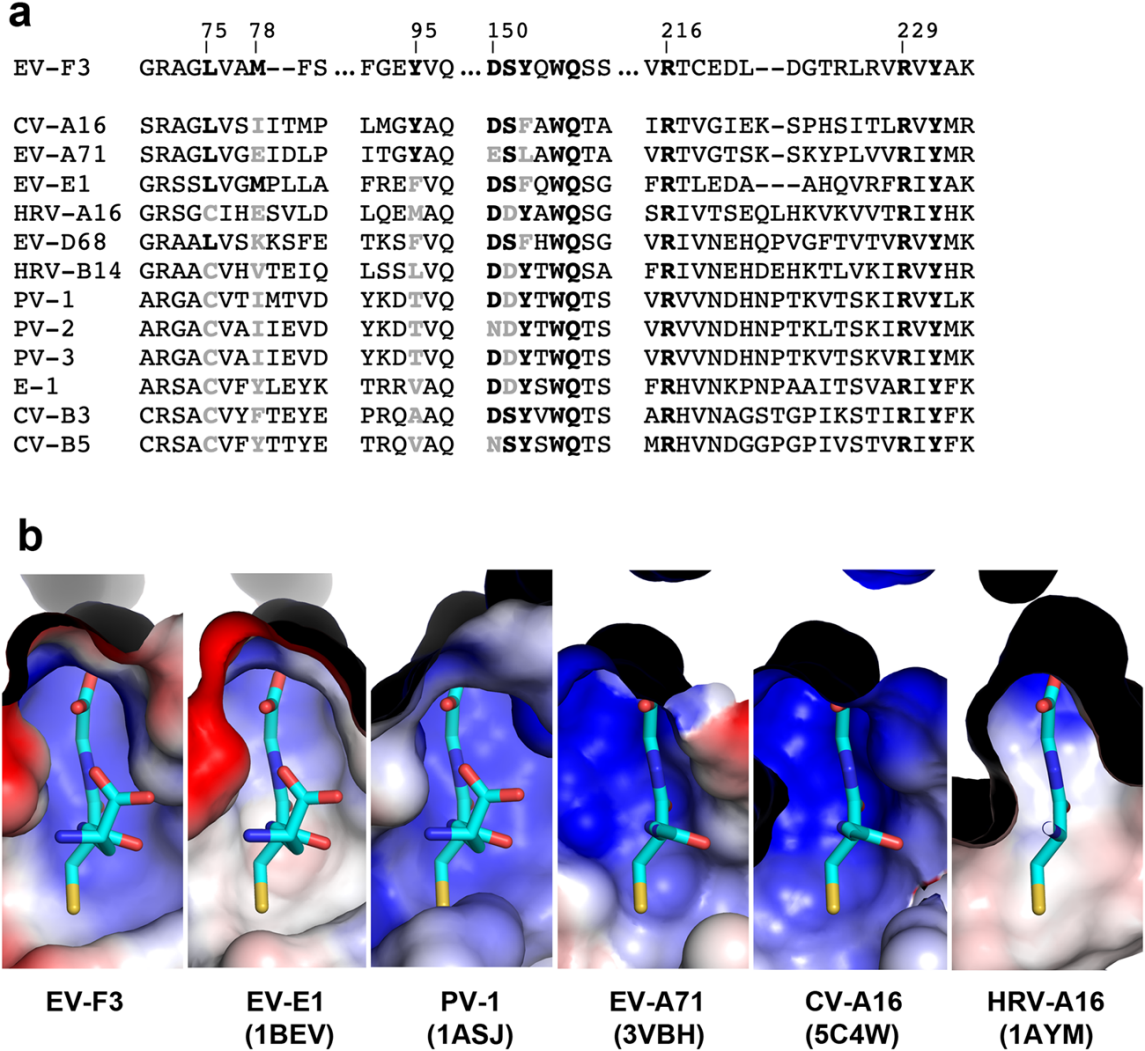
Site *I* is conserved for major enteroviruses. **a** Sequence alignment of VP1 for several major enteroviruses. Conserved GSH-interacting residues are in bold. **b** Electrostatic charge (±5 kT e^−1^) mapped onto the viral surface and coloured blue (positive) and red (negative), for selected enteroviruses, as labelled.

The *I* site comprises six segments of polypeptide chain contributed by two adjacent protomers, A and B. Three of these are from VP1 (protomer A), residues 75–79, 95–98 and 227–231 respectively, a fourth is from the C-terminus of VP3 (protomer A), and the fifth and sixth are from residues 150–155 and 216 of VP1 of protomer B (Fig. 1d). Numerous hydrophobic interactions, hydrogen bonds and salt bridges stabilise GSH binding (Fig. 3) and several interacting residues are conserved across enteroviruses (Fig. 4a). Notable are the salt bridges formed between the carboxy terminus of GSH and the side chains of VP1 R216 (protomer B) and VP1 R229 (protomer A), which form the core of a strong positive patch within the *I* site (Figs. 1d and 3). The CG portion of GSH anchors the two adjacent protomers via not only these salt bridges, but also by additional H-bonding interactions with Y231 of protomer A and the carbonyl oxygen of W154 of protomer B via a bridging water molecule. The main chain amide and carbonyl groups of the C residue of GSH form additional H-bonds with the side chain of the carbonyl oxygen of D150, and the side chain of Q155, respectively (both protomer B). Although the carboxylate group of the E residue of GSH makes no direct contacts with the *I* site, other portions of the residue contact D150 (protomer B) and, indirectly, R227 (protomer A) at the inter-protomer interface (Figs. 1d and 3). Given that for GSH the strongest interactions are formed by the C-terminus of the ECG tripeptide, the binding of CG is not unexpected, the preference for GSH, however, confirms that additional binding energy is contributed by residue E, although its side chain can assume multiple conformations (Fig. 2b, c).

### The GSH binding site is conserved and is occupied in several virus structures

The patch of positive charge within the *I* site is conserved across a panel of enteroviruses (note strict conservation in VP1 of R216 and R229 and the presence of either K or R at 233 in Fig. 4a) and although the width of the pocket varies, it remains sufficient to accommodate glutathione (if the flexible Glu side chain is allowed to rearrange, Fig. 4b). In several previously determined enterovirus structures, *I* site density has been observed and variously interpreted as water (EV-A71, 3VBH)^25^, chlorine (CV-A16, 5C4W)^26^, glycerol (HRV14, 4PDW)^27^ and glutamic acid (EV-F3, 5OSN)^7^. These previous interpretations sometimes fail to fully explain the observed density (Supplementary Fig. 3), although these densities also do not closely resemble GSH and their identities remain unknown.

### The GSH binding pocket also binds enterovirus inhibitors

**Fig. 5:**
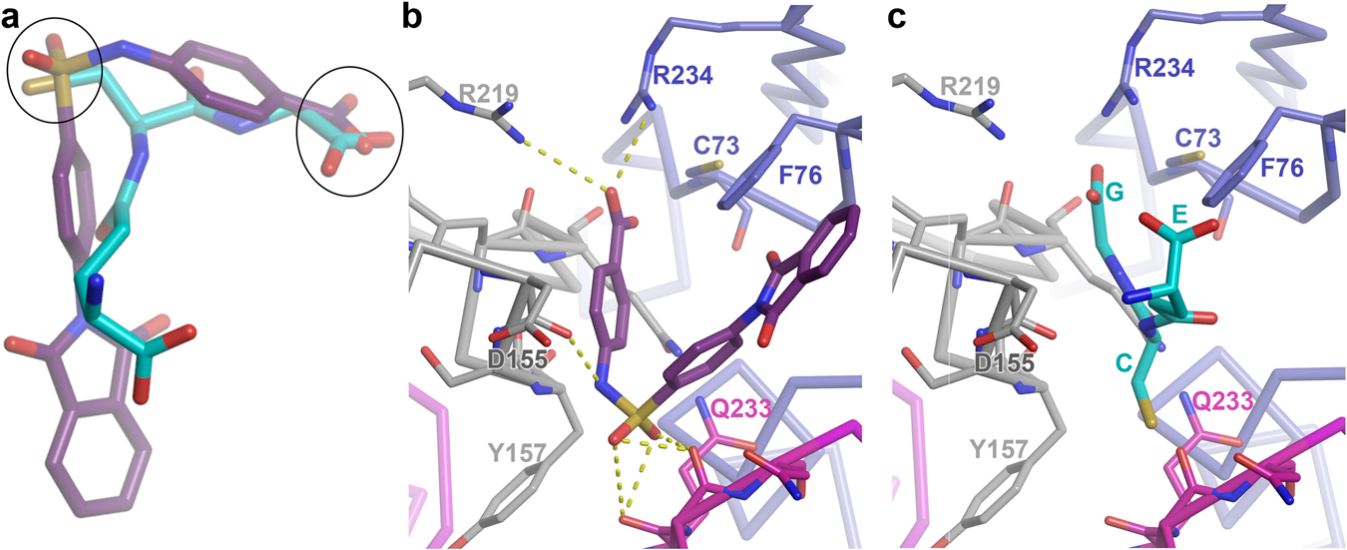
Compound-17 forms sub-optimal interactions when mapped onto site *I*. **a** GSH (cyan) superposed on compound-17 (purple) by matching surrounding protein structure^8^. The superpositions of a carboxylic acid group and a sulphur between the two are marked with ellipses. **b** Compound-17 (purple) bound to CVB3 (PDB: 6GZV)^8^. Protomer A VP1 is blue and VP3 magenta. Promoter B is in grey. **c** GSH positioned by superposition of the surrounding protein into CB-V3.

Very recently, ‘compound-17’, a benzene sulphonamide derivative, was identified as a CV-B3 inhibitor (Fig. 5a)^8^. Elaborations of this compound were identified with activity against a range of enteroviruses^8,28^, and electron cryo-microscopy revealed the approximate binding site at 4 Å resolution. Comparison of binding sites shows that GSH and compound-17 bind in the same pocket (Fig. 5), although the conformation and interactions of compound-17 are sub-optimal relative to GSH; as expected, given the much lower resolution of the analysis, there are common elements in the binding (Fig. 5).

## Discussion

Pan-enterovirus conservation of key residues within the *I* site suggests that it may have potential as a broad-spectrum druggable site. While such conservation encompasses enteroviruses that have been identified as GSH-independent, e.g. EV-A71, it is plausible that GSH can still exert a stabilising effect on the capsid, blocking the transition to the altered state (known as A-particle) that is thought to be a precursor to uncoating and infection^25,29^. In line with this, the antiviral activity of compound-17 is not impeded by increased intracellular glutathione levels, consistent with inhibition of a stage of the infection process which occurs before virus assembly in the cytoplasm, such as uncoating in the endosome. Compounds that increase the stability of the virus capsid may also be useful as vaccine-stabilising additives^23^. Indeed it has been found that oral poliovirus vaccine is stabilised by GSH^23^, and thus developing a more potent binder may be useful for the formulation of future virus-like-particle-based vaccines, including, but not limited to, PV^30^.

In summary, our structures explain how GSH facilitates initial enteroviral assembly into pentamers. The structural conservation of the *I* site suggests that this protomer glue is widely used, with natural molecules remaining associated at this site for many purified enteroviruses. Together with the recent studies on benzene sulphonamide derivatives^8,28^, it is clear that site *I* presents a druggable pocket, and our high-resolution results provide a firm basis for structure-led drug discovery.

## Methods

### EV-F3 production and purification

BHK-21 cells were grown to 80% confluency before infection with EV-F3 virus stocks in Glasgow modified Eagle’s medium (G-MEM BHK-21, Gibco), supplemented with 2% fetal horse serum (Thermo Fisher Scientific) and minimal essential medium non-essential amino acids (MEM NEAA, Gibco). Cells were incubated at 37 °C for 3 days before harvesting. Virus-infected media was first clarified, before overnight PEG-precipitation of virus particles. PEG-precipitated virus was then re-suspended in 50 mM HEPES pH 8.0, 200 mM NaCl, 0.5% NP40 buffer, and particles pelleted on a 30% sucrose cushion. The resulting pellet was re-suspended in minimal 50 mM HEPES pH 8.0, 200 mM NaCl, applied to a 15–45% w/v sucrose gradient and subjected to ultracentrifugation at 4 °C for 3 h, 105,000 *×* *g*. A clear band at approximately 30% sucrose concentration was observed, extracted and the presence of EV-F3 was confirmed by SDS-Page gel electrophoresis.

### PaSTRy

Solutions of EV-F3 and additive at either 100× or 1000× molar excesses (corresponding to 0.1 mM or 1 mM final concentration) were aliquoted into a 96-well PCR plate containing SYTO9 dye (Thermo Fisher Scientific) in triplicate with 50 mM HEPES, pH 8.0, 200 mM NaCl buffer, before centrifugation for 3 min at 4 °C. The assay was performed by heating samples in a Mx3005p qPCR machine (Agilent Technologies, USA) from 25 to 97 °C in 1 °C min^−1^ increments for 30 s in an expanding saw-tooth profile, a refinement of a previously described protocol^24^. Fluorescence changes were monitored at 25 °C with excitation and emission wavelengths of 492 and 517 nm, respectively.

### Infectivity assays

Assays were performed with BSR-T7 cells maintained in G-MEM BHK-21, supplemented with 5% fetal bovine serum (FBS, Life Technology) and MEM NEAA (Gibco) seeded into 96-well plates. For BSO treatment, cells were GSH starved for 48 h before washing and addition of EV-F3 and further 0.4 mM BSO (Sigma). Control plates were also seeded 48 h prior to infection. One hundred microlitres of tenfold serial dilutions of EV-F3 in serum-free G-MEM was added to washed, confluent cells. For BSO-treatment conditions, additional BSO was added to each of the EV-F3 dilutions. CPE could clearly be seen after 12 h (compared with negative controls). Eight replicates of each dilution were plated and were monitored over 2 days at 37 °C and 5% CO_2_. Wells were visually scored for virus growth at 17 h post infection (Supplementary Table 1). GSH depletion by BSO eventually disrupts cell integrity and thus it was not possible to maintain assays for more than 2 days.

### Crystallisation

Crystals were grown using previously reported protocols^7,31,32^. Briefly, sitting drop crystallisations were set up in 96-well trays using a Cartesian robot with a reservoir solution of 1.5 M ammonium sulfate and 0.1 M Tris at pH 8.5, each crystallisation drop containing 100 nL each of protein and reservoir solutions.

Crystals of approximately 50–80 μm diameter were soaked overnight with either (1) GSH; (2) CG; or (3) GSH/CG at 10 mM (thus for experiment 3 the total concentration of ligand was 20 mM) and then cryo-protected using sequential addition of glycerol from 10 to 25% (v/v). The compound to be soaked was maintained during the cryo-protection process.

### X-ray data collection

Diffraction data were collected mainly using automated X-ray centring and data collection at 100 K at I03 beamline, Diamond Light Source (Didcot, UK), with an X-ray wavelength of 0.976 Å. Diffraction images of 0.03° or 0.05° rotation were recorded on either a Pilatus3 6M, or Eiger2 X 16M detector (see Table 1) using a beam size of 80(H) × 20(V) µm^2^. A 0.0075 s or 0.01 s exposure time per 0.03 or 0.05° image with 100% beam transmission, under cryogenic conditions, enabled the collection of up to 180° of data before radiation damage was observed. Data from a single crystal were used for experiment (2) whilst data from two crystals were merged for experiments (1) and (3) (Table 1).

### X-ray data processing

Data were indexed and integrated using either the xia2-dials pipeline (experiment (3))^33,34^, or XDS^35^, before scaling and merging with AIMLESS (CCP4i)^36,37^. Structures were initially determined by rigid body fitting of the EV-F3 XFEL structure (PDB: 5OSN)^7^ using increasing resolution cut-offs in CNS^38^. Some unit cell shrinkage was observed relative to the room-temperature XFEL structure, consistent with these data being collected under cryogenic conditions^7^. Cyclic positional and B-factor refinement with strict NCS constraints was then performed with CNS, and models were improved with COOT^39,40^. For experiments 1, 2 and 3, respectively, the percentages of residues in the favoured region of the Ramachandran plot are 94.5%, 95.1% and 94.9%. If the allowed regions are included, these percentages rise to 98.6%, 99.2% and 99.2%, respectively. Ramachandran and rotamer outliers for experiment 2 are shown in Supplementary Fig. 2, confirming our modelling. Other statistics are provided in Table 1. All structural figures were constructed using PyMol^35^.

### Statistics and reproducibility

Infectivity assays were performed with eight repeats. PaSTRy assays were performed in triplicate, with mean values and standard deviations shown in Supplementary Fig. 1. Associated melting temperatures were determined as the temperature half way between the bottom and top of each Boltzmann sigmoidal fit in GraphPad Prism.

### Reporting summary

Further information on research design is available in the Nature Research Reporting Summary linked to this article.

## Supplementary information

Supplementary Information

Reporting Summary

## Data Availability

Coordinates and structure factors for EV-F3 under conditions (1), (2) and (3) are deposited in the PDB with accession codes 6T4C, 6T40 and 6T48, respectively.
