# Supplementary material for: Glutathione facilitates enterovirus assembly by binding at a druggable pocket
Source: Commun Biol. 2020 Jan 3;3:9. doi: 10.1038/s42003-019-0722-x (PMC6941975; doi:10.1038/s42003-019-0722-x)
Supplement: Supplementary file 1 — Supplementary Information [file 42003_2019_722_MOESM1_ESM.pdf]

a)

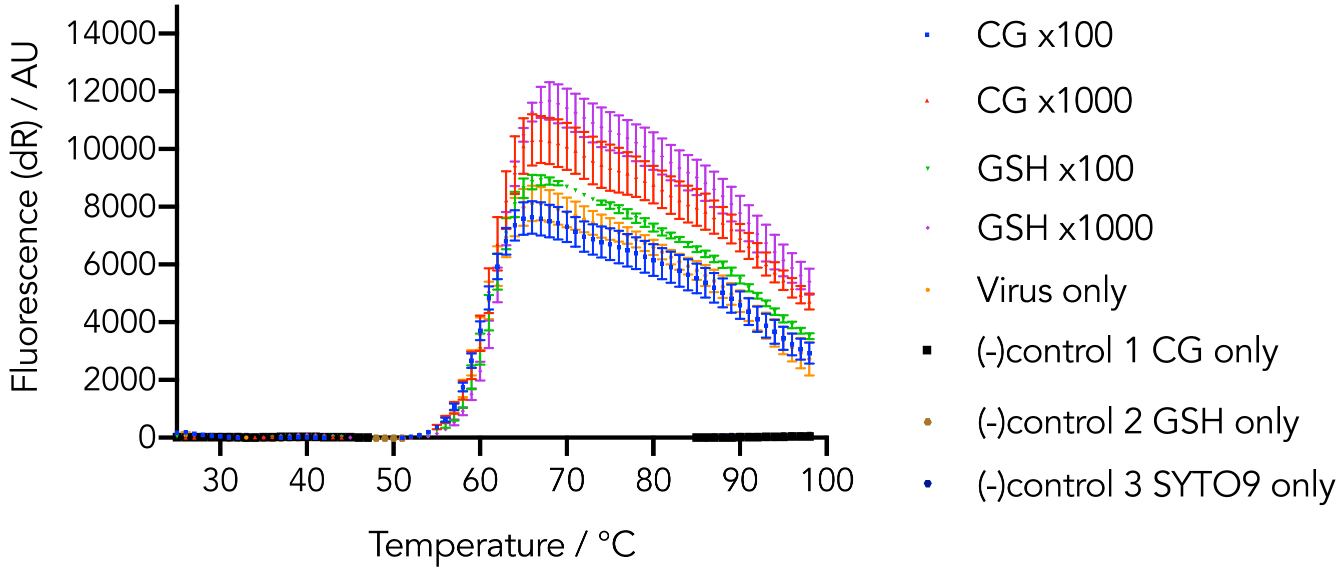

b)

	Tm (°C)	SD (±°C)
GC100	60.08	0.16
GC1000	61.06	0.22
GSH100	61.10	0.14
GSH1000	62.35	0.12
Virus only	60.40	0.20

Supplementary Figure 1 | (a) PaSTRy assay plot and table from 100x and 1000x molar excesses of CG or GSH to EV-F3. All experiments were performed in triplicate. Midpoints were determined using a Boltzmann fit (shown), generated using Prism (GraphPad Software, San Diego). (b) Table of midpoints (standard deviations derived from Prism).

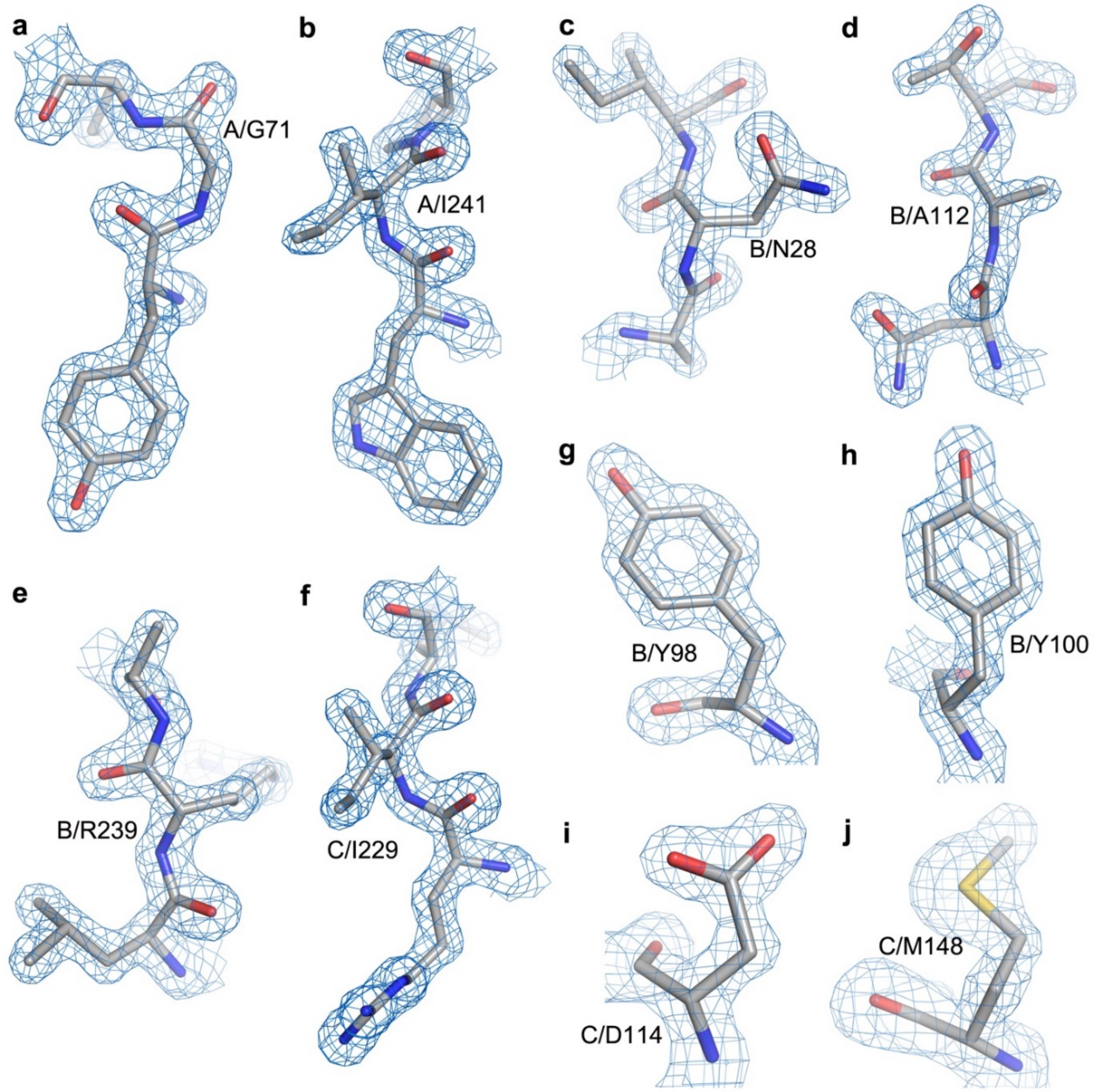

Supplementary Figure 2 | Electron density for outliers in either main-chain torsion angle or side-chain rotamer (for the CG soak, experiment 2). Residues G71 (**a**) and I241 (**b**) of VP1 (chain A), N28 (**c**), A112 (**d**) and R239 (**e**) of VP2 (chain B) and I229 (**f**) of VP3 (chain C) are the outliers in the Ramachandran plot, but have well defined density. The density is also well defined for the rotamer outliers, Y98 (**g**) and Y100 (**h**) of VP1, and D114 (**i**) and M148 (**j**) of VP3.

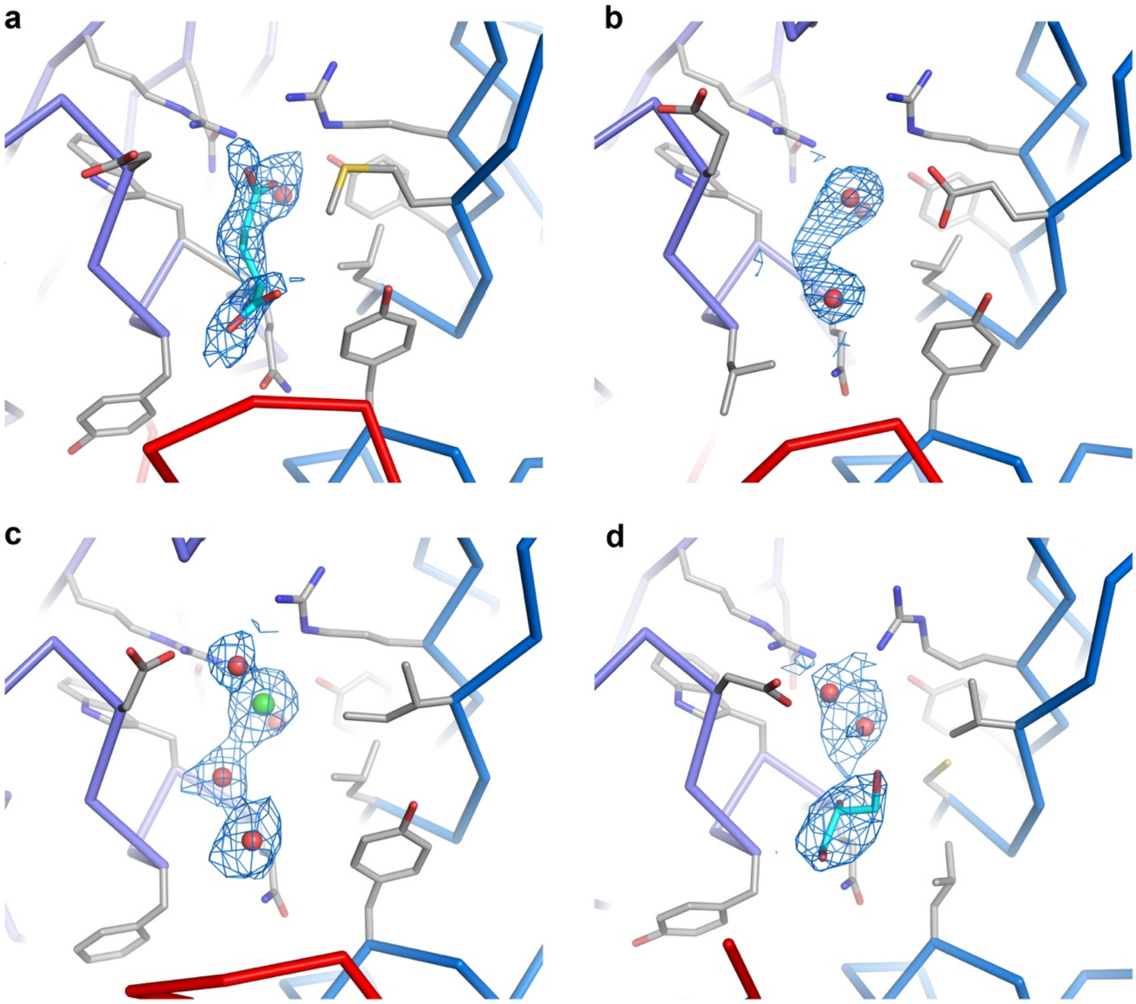

Supplementary Figure 3 | Previously observed electron density at the GSH binding site of some EVs. a, EV-F3 XFEL structure (PDB ID, 5OSN), where the density was modelled as a glutamic acid (cyan sticks). **b**, EV-A71 (PDB ID, 3VBH), modelled as waters. **c**, CV-A16 VLP (PDB ID, 5C4W), modelled as chlorine and water molecules. **d**, HRV-B14 (PDB ID, 4PDW), modelled as glycerol (cyan sticks) and water molecules. Capsid protein main-chain backbones are shown as thicker sticks with VP1 and VP3 in bright blue and red respectively, and the VP1 from the neighbouring protomer in pale blue. Side-chains are drawn as grey sticks, water and chlorine molecules as red and green spheres.

Supplementary Table 1 | CPE assay. Percentage of wells of BSRT7 cells showing CPE (percentage of wells showing CPE, out of 8) 17 hrs post infection (determined visually) at sequential EV-F3 dilutions.

EV-F3 DILUTION	(-)	10^{-1}	10^{-2}	10^{-3}	10^{-4}	10^{-5}	10^{-6}	10^{-7}	10^{-8}	10^{-9}	10^{-10}
**-BSO**	0.0	100	100	87.5	87.5	87.5	50.0	25.0	25.0	0.0	0.0
**+BSO**	0.0	100	62.5	37.5	12.5	0.0	0.0	0.0	0.0	0.0	0.0
